# COVID-19 School vs. Community-Based Outbreak Trends among New Jersey K–12 Schools during the 2020–2021 School Year

**DOI:** 10.3390/ijerph19159285

**Published:** 2022-07-29

**Authors:** Juhi Aggarwal, Maureen W. Gichura, Maryanne L. F. Campbell, Kimberly T. Nguyen, Derek G. Shendell

**Affiliations:** 1 New Jersey Safe Schools Program (NJSS), School of Public Health (SPH), Rutgers Biomedical and Health Sciences, Rutgers—The State University of New Jersey (NJ), Piscataway, NJ 08854, USA; ja880@sph.rutgers.edu (J.A.); mwg59@sph.rutgers.edu (M.W.G.); mlf159@sph.rutgers.edu (M.L.F.C.); ktn26@sph.rutgers.edu (K.T.N.); 2 Department of Biostatistics & Epidemiology, School of Public Health (SPH), Rutgers Biomedical and Health Sciences, Rutgers—The State University of New Jersey (NJ), Piscataway, NJ 08854, USA; 3 Department of Environmental & Occupational Health & Justice, School of Public Health (SPH), Rutgers Biomedical and Health Sciences, Rutgers—The State University of New Jersey (NJ), Piscataway, NJ 08854, USA

**Keywords:** COVID-19, community-based outbreak, primary schools, secondary schools, school-based outbreak, school safety

## Abstract

Identifying potential rapid methods to track COVID-19 trends within schools has become a necessity in understanding how to provide both education and maintain health and safety during a pandemic. This study examined COVID-19 trends and sociodemographic information in New Jersey (NJ) schools during the 2020–2021 school year. A database was compiled for this study in Microsoft Excel using various state and federal resources. Data used in the study are a combination of extracted data from weekly NJ Spotlight reports, weekly NJ COVID-19 Activity Level Index (NJ CALI) reports, and reports of school-based outbreaks via the NJ Department of Health (NJDOH). In 2020–2021, in NJ K–12 schools, the NJDOH defined a school-based outbreak incidence as two or more students and/or adult staff with a laboratory-confirmed positive molecular test for COVID-19 based on transmission occurring on campus. Data were organized into six regions across 21 counties within NJ (3–4 counties per region per NJDOH). COVID-19 trends in NJ schools mirrored trends in their districts, i.e., communities, within the state’s region; noticeably, there were consistently high trends during the winter holiday season (November 2020–January 2021). The cumulative number of incidences of school-based outbreaks remained relatively low but, nevertheless, increased throughout the 2020–2021 school year. This study recommends increased accessibility to COVID-19 reports for school and public health officials, and in the future for data to be reported to identify rates of transmission of other communicable diseases within K–12 students, and to further reinforce established mandates and other preventative measures in public while traveling during holiday seasons.

## 1. Introduction

The World Health Organization (WHO) confirmed the novel COVID-19 outbreak of Wuhan, China, as a global pandemic on 11 March 2020. After examining the number of cases outside China, the WHO was deeply concerned about its rapid growth and severity, and the alarming levels of preventative action required to properly slow the spread of the virus [1]. In efforts to slow the spread, national and state governments such as in New Jersey (NJ) took many precautions during the following weeks to ensure the safety of their citizens—including mandates regarding wearing masks, social or physical distancing, and working and learning from home as well as lockdowns. The United States also enacted national lockdowns while eventually allowing state governments to impart specified mandates and lockdowns—even when these actions restricted everyday activities and transitioned many of them to become virtual, including education. As the pandemic progresses, public health officials are challenged to consistently provide safety guidance and strategies to lawmakers to navigate life safely.

While in-person instruction is important for young people, school-aged children were considered a high-risk population for the COVID-19 virus. The U.S. Centers for Disease Control and Prevention (CDC) recognizes how important in-person instruction is and has cited multiple studies that keep children, teachers, and staff safe. This includes the administration or implementation of COVID-19 vaccines, masks and physical distancing requirements, screening testing, proper ventilation improvements, handwashing, keeping children that are sick at home, contact tracing, and adjusting meal schedules and physical activity schedules (physical education, sports, other activities). Though these preventative measures have been put in place, COVID-19 outbreaks still occur within schools, early care, and education programs [2,3,4].

Identifying potential rapid methods to track COVID-19 trends within schools has become a necessity to understand how education can safely be provided during a pandemic. The mission of the NJ Safe Schools Program within the state of NJ is “to assist schools in reducing risk to occupational safety and health hazards in secondary school and work microenvironments in which NJ adolescents spend time” [5]. The purpose of the present study was to examine COVID-19 trends among NJ schools during the 2020–2021 school year. We also wanted to integrate sociodemographic information in our analysis to give a holistic perspective on COVID-19 outcomes in K–12 students.

## 2. Materials and Methods

A database was compiled for this study in Microsoft Excel (Microsoft Corporation, Redmond, WA, USA) to input, organize, format, and observe data extracted from various state [6,7,8] and federal resources [9,10].

### 2.1. COVID-19 Surveillance Data

Data presented in the weekly NJ Spotlight data were extracted, including from weekly NJ COVID-19 Activity Level Index (NJ CALI) reports statewide and reports of school-based outbreaks.

In 2020, as used through 16–17 June 2022, in NJ, NJ Department of Health (NJDOH) defined NJ CALI—for each of six regions and then statewide (as average of averages)—as the average of the values for each indicator. The NJ CALI values on a 1–4 scale were based on the activity range each indicator’s value fell into. The defined indicators were three 7-day averages: COVID-19 case rate, defined as the number of positive PCR-confirmed tests per 100,000 population based on the patient specimen collection date; COVID-like illness, defined as fever and cough or dyspnea (shortness of breath, difficulty breathing, etc.) or the presence of coronavirus diagnosis codes but influenza-like illness (influenza, parainfluenza and RSV or respiratory syncytial virus) was excluded; and, COVID-19 PCR-based test positivity as a percent [6,8,11].

In 2020–2021, in NJ K–12 schools, NJDOH defined a school-based outbreak incidence as ≥2 students and/or adult staff with a laboratory-confirmed positive molecular test for COVID-19 based on transmission occurring on campus. (In 2021–2022, the NJDOH changed the definition to ≥3). These were made accessible via the NJDOH website [6,8].

**Figure 1:**
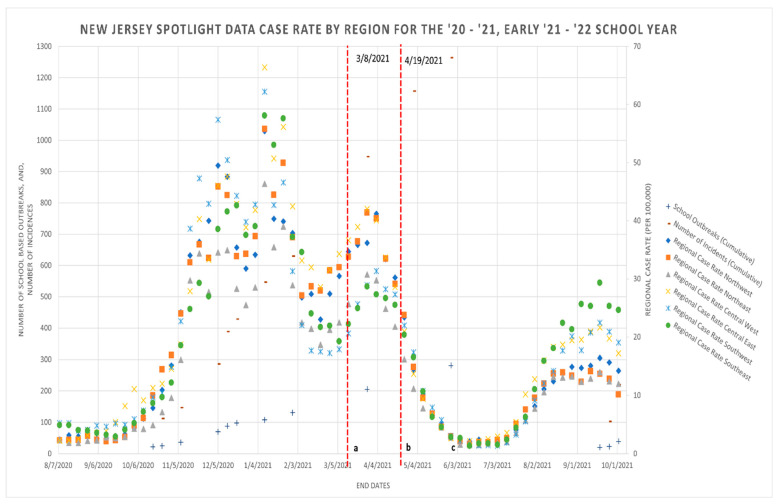
Data acquired from New Jersey Department of Health (NJDOH) and NJ Spotlight database and media report summaries (8 August 2020–2 October 2021) ^a–c^. ^a^ NJDOH announces all adult and day care workers in the state are eligible to have free COVID-19 vaccines starting 8 March 2021 [12,13]. ^b^ Governor Murphy announces all New Jersey residents aged 16 and over are eligible to receive the COVID-19 vaccine starting 19 April 2021 [7,13]. ^c^ After Memorial Day of 2021 (Monday, 31 May 2021), schools were preparing for summer recess and children were going outside less around this period.

**Figure 2:**
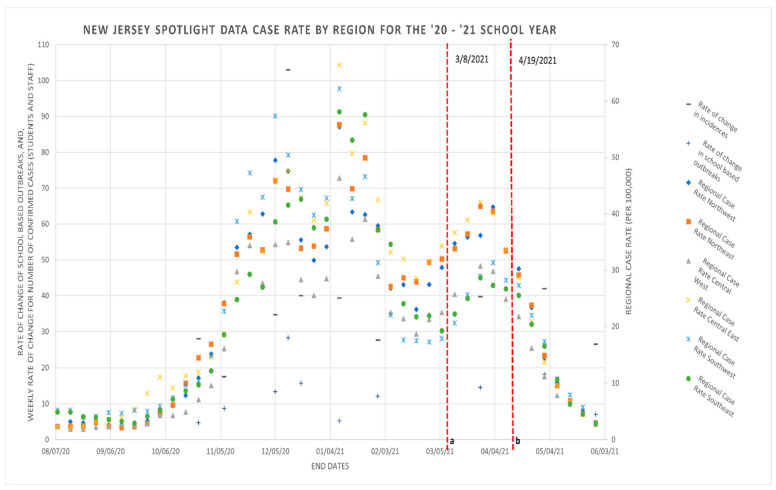
Data acquired from New Jersey Department of Health (NJDOH) and NJ Spotlight database and media report summaries with rate of changes in incidences and school-based outbreaks, respectively (8 August 2020–5 June 2021). ^a^ NJDOH announces all adult and day care workers in the state are eligible to have free COVID-19 vaccines starting 8 March 2021 [12,13]. ^b^ Governor Murphy announces all New Jersey residents aged 16 and over are eligible to receive the COVID-19 vaccine starting 19 April 2021 [7,13].

The data were organized into the 21 counties and then into 6 regions within NJ. Regions are classified by the NJDOH based on the location of counties in the state of NJ [8,11]. Counties in each defined NJ public health region (acronyms used in Figure 1 and Figure 2 only) are as follows: Northwest (NW): Morris, Passaic, Sussex, Warren; Northeast (NE): Bergen, Essex, Hudson; Central West (CW): Hunterdon, Mercer, Somerset; Central East (CE): Middlesex, Monmouth, Ocean, Union; Southwest (SW): Burlington, Camden, Gloucester, Salem; Southeast (SE): Atlantic, Cape May, Cumberland.

Variables for which we identified data available from NJDOH [6,8] were: regional case rate (RCR); sociodemographic data from census per county; school year; quarter and semester of school year; weekly start and end dates; state county; percent positivity (the percentage of total positive polymerase chain reaction (PCR) tests out of all tests performed); reported CALI scores; COVID-19 activity level (low risk, moderate risk, high risk, very high risk); number of school-based outbreaks; number of cases/infections linked to school-based outbreaks; number of infections within school districts; remote learning status (in-person, remote, or hybrid learning); vaccine availability according to policy and age groups. Case rate was a calculated proportion based on the population of daily new COVID cases, confirmed by PCR results, for every 100,000 people. Case rate was further monitored as a 7-day average by the first positive specimen collection date.

NJDOH changed the system in which school-based outbreaks were tracked between September and October of 2021, effective late October 2021. Furthermore, Hurricane Ida impacted Labor Day Weekend of 2021, which either caused a delayed reopening of in-person schooling and/or use of remote learning for initial week(s) of the school year.

### 2.2. Sociodemographic Data

Sociodemographic data for the state of New Jersey were obtained from the American Community Survey (ACS) of 2019 and 2020 regarding “age and sex” and “race”, respectively [9,10]. These data were public (no copyright permission needed) and available for incorporation into secondary analyses. The ACS data included race, age (by groups such as 5–9 years, 10–14 years, 15–17 years, 18–19 years, and 20–24 years for children and young adults), and students in public schools versus private schools. The ratio of students in public schools versus private schools was then calculated by age (for five age groups representing pre-K to 12th grade). According to the U.S. Office of Management and Budget, race can be self-reported using five groups, with a sixth category of "some other race," which can include people who identify their ethnic origin as Hispanic, Latino, or Spanish, regardless of their race [8,10]. Other race is a combination of American Indian and Alaska Native, Native Hawaiian and other Pacific Islander, and “some other race” as aforementioned.

## 3. Results

### 3.1. COVID-19 Surveillance Data

Figure 1 includes surveillance data obtained from the NJDOH tracking COVID-19 case rates (per 100,000) by region, number of school-based outbreaks, and number of incidences reported during the 2020–2021 school year and from early in the 2021–2022 school year.

The trends of school-based outbreaks and incidences coincide with the trends seen in regional cases rates. Specifically, the increases in the cumulative number of incidences and outbreaks reported from November 2020 to February 2021 coincide with the celebrations of the Thanksgiving/winter holiday season and how traveling may have possibly impacted these case numbers. A break in this trend is the major difference or visibly apparent spike in the number of school-based incidences recorded between 5 March–3 June 2021. Indeed, this can be observed in both Figure 1 and Figure 2.

Figure 2 includes surveillance data obtained from the NJDOH tracking COVID-19 case rates (per 100,000) by region, rate of changes in the number of school-based outbreaks, and incidences reported during the 2020–2021 school year. The present study’s analysis tracks the rates of changes in school-based outbreaks and incidences among students and staff. Finally, it is worth noting how the Central East region of NJ had some of the highest regional case rates and the Central West region mostly had lower regional case rates.

The trends of school-based outbreaks and incidences seem to coincide with the trends seen in regional cases rates, e.g., the spike in the rate of incidences and outbreaks during November 2020–February 2021 (given the travel and celebrations during the Thanksgiving/winter holiday season) follows the regional case rates. Nonetheless the highest incidence within this time period is lower than the regional case rate in the Central East region according to the State of NJ [6]. A break in this trend and a major difference is a spike in the number of school-based incidences between 5 March–3 June 2021.

### 3.2. Sociodemographic Data

**Figure 3:**
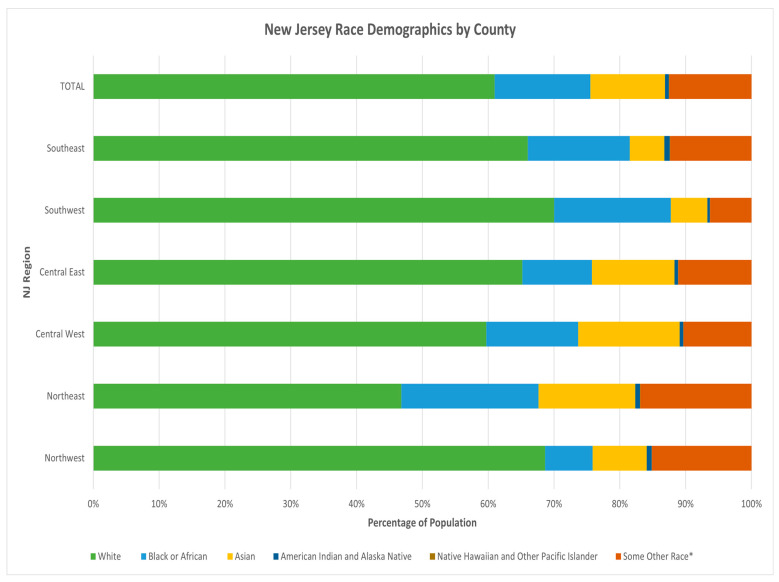
Stacked bar graph of race by county in the state of New Jersey compared to percentage of population. * U.S. Office of Management and Budget permits race to be self-reported in five (5) groups with a sixth category of “some other race [10]”.

Race is represented via a stacked bar graph detailing race per region (see Figure 3). Within the state of NJ, the overall race self-identification percentages are as follows: 61.0% White, 14.5% Black or African, 11.3% Asian, 0.6% American Indian and Alaskan Native, 0.04% Native Hawaiian and other Pacific Islander, and 12.5% Other race. A stacked bar graph shows the percentage of the specific race within a region compared to the other races. For example, of the entire population of the Southwest region of NJ, 70.0% self-identify as white. Of the entire population of the Northeastern region of NJ, 46.8% self-identify as white and over 20.8% self-identify as Black or African (the highest percentage in the state). The highest percentage of people who self-identify as Asian (15.4%) was in the Central West region of NJ.

The ACS divided age groups as follows: 5–9 years, 10–14 years, 15–17 years, 18–19 years, and 20–24 years [9]. Ratios of students in public to private schools were calculated and tabled for comparisons of regions and counties (as shown in Table 1). The Southeast region had the highest ratio of public to private schools for all age groups except for those between 20 and 24 years where the Southwest region had the highest ratio. The lowest ratios were observed as follows: 5–9 years, 10–14 years, and 15–17 years within the Central East region, and 18–19 years and 20–24 years in the Central West region.

Statewide, across K–12 public and private schools, about 35% of students are 10–14 years old (overlapping primary and secondary school grades), 33% are 5–9 years old (primary schools), 21% are 15–17 years old (secondary schools), and 11% are young adults, ages 18–19 (secondary schools). Among the six defined regions of NJ, the Central East and Northwest regions have the largest proportion of K–12 students and the Southeast region has the smallest proportion of K–12 students, overall and by the four defined age groups, within the state of NJ in both public and private schools.

## 4. Discussion

Overall, this secondary analysis of these data from state and federal agencies for the 2020–2021 school year, a specific part of the ongoing COVID-19 pandemic, suggested a relative increase in the rate of change in school-based outbreak incidence in late fall 2020 (November–mid-December), which coincided with an increase in regional case rates across NJ counties in early winter (December 2020–early February 2021).

This secondary analysis could not analyze the results by socioeconomic status indicators at region or county levels due to the lack of data available at school district or school levels. Local school districts only had prior school years 2018–2020 data available on their website, due to staffing and reporting changes made because of the COVID-19 pandemic in the 2020–2021 school year, resulting in the delay in the entirety of the 2020–2021 school year data. This also affected the demographic aspect of this secondary analysis, especially when looking at race. The ACS data included county level data, and with approximately 2500 schools and 600 school districts in the state of New Jersey, calculating race per county may not be the best indicator for a specific school/school district. In this study, overall, regarding race, individuals who self-identified as white composed the majority of the NJ K–12 school student population, and the highest sub-population of K–12 students were those 10–14 years of age. Future K–12 surveillance should examine disparities.

### 4.1. Strengths

Most of the data were easily accessible through weekly reports archived and recorded for the 2020–2021 academic year. The data were assumed to be valid and reliable because of the reputable sources from which data were extracted, including the online NJDOH data dashboard and ACS (the decennial U.S. Census). COVID-19 case rates per region were accessed from a reliable and accurate source, i.e., taken from the NJDOH data dashboard, and were calculated as proportions of the population. Real-time PCR results for COVID-19 were also gathered from the NJDOH data dashboard. These data were obtained from laboratory-based transmissions submitted by acute care, commercial, and public health laboratories to the Communicable Disease Reporting and Surveillance System [6,8,9]. ACS data were effective in supplying sociodemographic data for analysis. The age groupings within the data were significant in accommodating our research objective for analyzing K–12 school-aged children. The separation of private schools and public schools was also potentially beneficial. This study could initially consider available resources even without the ability to directly collect and analyze data on socioeconomic status-related factors at individual or school or district levels. Additionally, private schools and public schools had both different expectations and internally/independently determined versus locally determined policies for safety and health, respectively. The New Jersey Safe Schools team is composed of individuals specializing in multiple public health disciplines. The process allowed open dialogue and different perspectives and interpretations of the data as assimilated by the State of New Jersey and other government entities from K–12 schools and the public.

### 4.2. Limitations

Tracking COVID-19 trends proved to be a challenge. Due to the novelty of COVID-19 at the time, the enforcement of reporting and monitoring efforts was less strict during the 2020–2021 school year compared to the updated tracking system and definitions used in the 2021–2022 school year. Cases and incidences of school-based outbreaks were hard to access due to the NJDOH website updating weekly, without a standing week-to-week archive of data either statewide or by county. In other words, new data were added on to, in a cumulative manner, past county/school-based information. Ideally, in future potential epidemics or pandemics, data should be reported in real time every week, if not more frequently, even daily, in a publicly-available format. This is in part because of the NJDOH changing the system used to track school-based outbreaks between September and October of 2021, effective late October 2021 [6].

There is the potential for loss of data because of Hurricane Ida’s impact on the Labor Day Weekend of 2021, which either caused a delay in the reopening of schools in-person and/or the use of remote learning for the initial week(s) of the school year. It must also be noted that our study cannot compare and contrast trends observed in data available within the State of New Jersey with any other U.S. state (given potentially similar or different definitions of school-based outbreaks, as well as vaccine, testing, and/or mask mandates) or the U.S. overall (given the variety of definitions of school-based outbreaks and data collection methods in the absence of a federally-required protocol and the aforementioned COVID-19-related mandates). Definitions of cases and infections within school-based outbreaks were defined as staff, teachers, and students infected, so students were not isolated when information was reported in NJDOH weekly updates.

Sociodemographic data from the ACS did not provide information on the sex for each of the targeted age groups analyzed in this study. The ACS data only focused on adults aged 18–24 years and older, whereas this study on K–12 schools included age ranges up to age 21. Furthermore, the data from ACS did not identify the race or ethnicity (Hispanic/non-Hispanic) of age groups. Additionally, utilizing self-reported racial identity data leaves room for measurement error. We were limited to ACS data because of the lag time between local-to-state-to-federal educational data reporting, as well as the impact of COVID-19 on school districts and schools. This study could only initially consider demographic factors without the ability to directly collect and analyze data at the individual or school or district levels.

## 5. Conclusions

The purpose of the present study was to examine COVID-19 trends in New Jersey (NJ) schools during the 2020–2021 academic year. COVID-19 trends in NJ schools mirrored the COVID-19 trends within the state’s regions. The winter holiday season of November 2020–January 2021 proved to affect both school and regional case rates. COVID-19 trends during the holiday seasons were consistently high within schools. The Central East region of NJ had the highest regional case rates, and the Central West region of NJ had the lowest during this study’s time period according to the State of NJ [4]. This study documented how the cumulative number of incidences of school-based outbreaks remained relatively low but, nevertheless, increased throughout the 2020–2021 school year.

### Recommendations for Future Research, Policy, and Practice

The NJDOH could make COVID-19 reports more accessible to public health officials within local health departments and at universities for further research efforts. In future potential epidemics or pandemics, data should be reported in real time every week, if not more frequently, even daily, in a publicly-available format. Given the largest sub-population of NJ K–12 students is 10–14-year-olds (3rd–8th grade), research is recommended to identify the rates of transmission of communicable diseases within this age group. As children are an at-risk population for COVID-19, mandates and policies to prevent the spread of COVID-19 should be reinforced in the Central East region (as this is the region with the highest regional case rates during this study’s time period according to the State of NJ) [6]. Policies and future protocols should be further reinforced when establishing mask mandates and other protective measures in the public traveling during holiday seasons. This research also suggests, with a longer time frame, to examine how vaccination can impact case numbers. There is hope of a future expansion in classifying gender identity and racial/ethnic groups for the K–12 age groups in the census for further analysis of occupational safety and health hazards. For example, regions with a larger minority composition are at higher risk of health disparities juxtaposed with lower health care coverage. One goal is to have health equity throughout regions for children regardless of their ethnic/race with the resources state governments are able to provide—especially through their schools. The information included in this study provides the possibility of analyzing socioeconomic data within the regions of New Jersey and how that can potentially impact government protocol and public health practices.

## Figures and Tables

**Table 1:** Ratios of K–12 public school students to private school students, by age, in the State of New Jersey (NJ). These calculated ratios are based on data reported in the American Community Survey (based on U.S. Census 2020) as of the end of the 2019–2020 school year [9] (Please also see Supplementary Table S1). Values in bold are the six regions within the State of NJ and for NJ overall.

	5–9 Years	10–14 Years	15–17 Years	18–19 Years	20–24 Years
**Northwest**	**8.79**	**12.38**	**9.26**	**3.15**	**2.93**
Morris County	9.91	15.29	10.80	1.81	2.02
Passaic County	8.69	8.99	6.16	6.33	3.78
Sussex County	8.84	30.62	23.75	18.43	4.47
Warren County	5.61	16.22	27.04	1.70	4.31
**Northeast**	**9.63**	**10.11**	**7.82**	**2.83**	**1.98**
Bergen County	6.05	7.54	6.33	3.55	1.76
Essex County	21.71	13.31	9.42	2.58	2.98
Hudson County	9.97	11.90	9.33	2.28	1.54
**Central West**	**9.08**	**9.12**	**7.62**	**2.56**	**1.75**
Hunterdon County	20.82	11.33	7.16	10.32	1.25
Mercer County	11.07	10.00	11.58	2.29	1.47
Somerset County	6.57	7.79	5.68	2.33	2.82
**Central East**	**4.21**	**6.10**	**6.03**	**3.91**	**2.29**
Middlesex County	7.41	13.56	14.39	9.62	2.97
Monmouth County	5.25	9.74	5.71	2.14	1.29
Ocean County	1.32	1.80	2.20	2.52	1.69
Union County	17.55	18.80	14.20	3.46	4.66
**Southwest**	**7.45**	**9.35**	**6.87**	**5.48**	**3.68**
Burlington County	6.63	11.75	7.60	4.20	2.93
Camden County	8.77	6.68	6.69	5.18	4.33
Gloucester County	5.68	14.93	6.36	7.67	4.02
Salem County	90.28	8.23	6.38	18.49	4.29
**Southeast**	**12.62**	**20.62**	**13.44**	**7.04**	**3.46**
Atlantic County	8.22	19.35	10.67	13.99	4.48
Cape May County	35.78	17.45	40.60	1.00	1.47
Cumberland County	21.50	24.69	14.85	5.52	2.80
**Statewide/State of NJ**	**6.79**	**8.82**	**7.36**	**3.48**	**2.38**

## Data Availability

This study’s data are secured on computers per the IRB approved stewardship of the New Jersey (NJ) Safe Schools Program and/or are publicly available from the NJ Department of Education, the NJ Department of Health, and/or the U.S. Census. Datasets used and analyzed during the current study are available from the corresponding author upon reasonable request.

## Supplementary Materials

The following supporting information can be downloaded at: https://www.mdpi.com/article/10.3390/ijerph19159285/s1, Table S1: Numbers of K–12 public school and private school students in the State of New Jersey (NJ) as reported in the American Community Survey (based on U.S. Census 2020) as of the end of the 2019–2020 school year [9].
