# Supplementary material for: COVID-19 School vs. Community-Based Outbreak Trends among New Jersey K–12 Schools during the 2020–2021 School Year
Source: Int J Environ Res Public Health. 2022 Jul 29;19(15):9285. doi: 10.3390/ijerph19159285 (PMC9367846; doi:10.3390/ijerph19159285)
Supplement: Supplementary file 1 [file ijerph-19-09285-s001.zip › ijerph-1838039-supplementary.pdf]

International Journal of
Environmental Research and Public Health

Supplementary Materials

Table S1. Numbers of K-12 public school and private school students in the State of New Jersey (NJ) as reported in the American Community Survey (based on U.S. Census 2020) as of the end of the 2019–2020 school year [9].

	Number of Public School Students by Age					Number of Private School Students by Age				
	5–9 Years	10–14 Years	15–17 Years	18–19 Years	20–24 Years	5–9 Years	10–14 Years	15–17 Years	18–19 Years	20–24 Years
**Northwest**	**59196**	**75144**	**43528**	**20848**	**25481**	**6731**	**6069**	**4700**	**6610**	**8709**
Morris County	22983	28674	18564	7311	9469	2320	1875	1719	4038	4688
Passaic County	26123	31847	16214	9737	10244	3007	3541	2631	1539	2712
Sussex County	6053	8574	5154	2249	3474	685	280	217	122	777
Warren County	4037	6049	3596	1551	2294	719	373	133	911	532
**Northeast**	**123458**	**132269**	**74762**	**31765**	**43209**	**12815**	**13086**	**9565**	**11208**	**21864**
Bergen County	44533	50067	31104	14023	18660	7356	6640	4913	3955	10588
Essex County	45342	51688	26645	10470	14858	2089	3882	2829	4058	4992
Hudson County	33583	30514	17013	7272	9691	3370	2564	1823	3195	6284
**Central West**	**40500**	**45416**	**28931**	**16809**	**17762**	**4462**	**4978**	**3798**	**6575**	**10123**
Hunterdon County	5830	6650	4621	2178	1412	280	587	645	211	1130
Mercer County	17694	20566	12561	9808	9870	1598	2056	1085	4291	6694
Somerset County	16976	18200	11749	4823	6480	2584	2335	2068	2073	2299
**Central East**	**126921**	**140895**	**84282**	**41016**	**52998**	**30144**	**23083**	**13984**	**10477**	**23110**
Middlesex County	41111	47684	28317	19096	22044	5549	3516	1968	1986	7421
Monmouth County	31727	31263	21021	7852	9918	6038	3211	3680	3675	7661
Ocean County	22023	25950	15308	6968	9281	16730	14441	6953	2766	5506
Union County	32060	35998	19636	7100	11755	1827	1915	1383	2050	2522
**Southwest**	**67766**	**71754**	**43034**	**18070**	**26565**	**9093**	**7673**	**6262**	**3300**	**7221**
Burlington County	21295	24767	14598	5508	8775	3211	2108	1920	1311	2991
Camden County	28437	27151	16395	6448	9761	3241	4065	2451	1244	2254
Gloucester County	14784	16691	9845	5430	6651	2605	1118	1547	708	1655
Salem County	3250	3145	2196	684	1378	36	382	344	37	321
**Southeast**	**25772**	**32881**	**16070**	**8166**	**8588**	**2042**	**1595**	**1196**	**1160**	**2481**
Atlantic County	12364	16928	8575	5414	6146	1504	875	804	387	1371
Cape May County	4616	4398	2639	335	735	129	252	65	335	500
Cumberland County	8792	11555	4856	2417	1707	409	468	327	438	610
**Statewide/State of NJ**	**443613**	**498359**	**290607**	**136674**	**174603**	**65287**	**56484**	**39505**	**39330**	**73508**
